# Te Whiringa Ora: person-centred and integrated care in the Eastern Bay of Plenty, New Zealand

**DOI:** 10.5334/ijic.2242

**Published:** 2015-09-23

**Authors:** Peter Carswell

**Affiliations:** School of Population Health, University of Auckland, Auckland, New Zealand

**Keywords:** care coordination, chronic care, integrated care, self-management, telehealth

## Abstract

Te Whiringa Ora is a community-based programme in New Zealand that facilitates interdisciplinary care for patients and their family. It targets those with a chronic disease whom have high inpatient admissions or emergency department presentations. It is based in a rural part of New Zealand that has a large indigenous population, and a relatively high level of social deprivation. The programme makes use of culturally appropriate care coordinators, and uses telephone support and tele-monitoring to aid self-management. The programme has been running for three years and has shown a reduction on hospital presentations, as compared to an equivalent population (not enrolled in the programme). This case study outlines the programme, and focuses specifically on the implementation processes, and lessons learnt.

## Part 1: the model of integrated care

### Background

Te Whiringa Ora^1^ (TWO) was established in February 2011 as a response to a call (and a refocusing of existing money) from the New Zealand government for the development of integrated primary care programmes that delivered ‘Better, Sooner, and More Convenient (BSMC)’ services [1]. The Better, Sooner, and More Convenient business case saw three existing Primary Health Organisations across the region of the Eastern Bay of Plenty merge into one structure, Eastern Bay Primary Health Alliance (EBPHA). This provided the critical mass and resources needed to invest in a programme that provided another way of supporting people with complex chronic illness(s). The alternative option that existed prior to this initiative was a general practice-based programme called ‘Care Plus’, in which practices received funding for patients to have a free visit four times a year to help improve the management of their long-term conditions (client eligibility based on people having two or more chronic conditions).

While the programme is funded by the Eastern Bay Primary Health Alliance, it was designed and implemented by Healthcare of New Zealand (HCNZ). Healthcare of New Zealand is the largest provider of community-based health and disability support in New Zealand, and one of the partners in the Eastern Bay Alliance.

The Te Whiringa Ora service is an integrated care service based in the community that facilitates interdisciplinary care to provide a web of care around patients (and their whānau) who are identified as having complex, long-term health needs and are high users of hospital services with the intention of improving their self-management. It works across general practices, hospital, community service providers and iwi providers.^2^ The purpose of the programme is to provide a responsive, coordinated and seamless delivery of services for patients and whānau^3^ incorporating Whānau Ora^4^ principles that consider physical, mental, emotional, spiritual and social characteristics of the patient and their whānau.

The objectives of the programme are wide ranging and include:

- improve access to primary, secondary and community health care.
- Provide seamless access to quality health services that meet clinical, social and cultural needs.
- Reduce disparities in health outcomes.
- Contribute to improving primary care management of chronic and long-term conditions.
- Support the health outcome priorities for Eastern Bay of Plenty.
- Reduce preventable hospital admissions and hospital length of stay.
- Increase pro-active intervention or delay deterioration which results in increasing levels of care and acute admissions.
- Provide a holistic patient-centred and Whānau ora approach to care.
- Educate service users and their whānau in self-management of chronic care lifestyle changes.

### Client group

**Figure 1.**
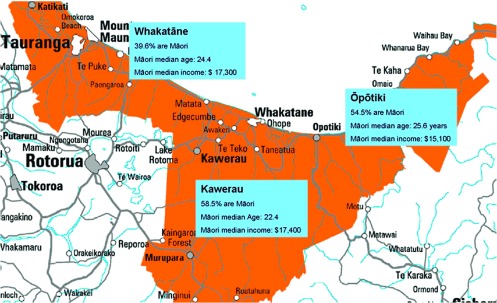
Eastern Bay of Plenty, New Zealand

Clients for Te Whiringa Ora come from across the Eastern Bay of Plenty in New Zealand (Figure 1). This area has approximately 50,000 people living in it across a wide geographic area, diverse in culture and make up. The population is bi-cultural, with 48 identifying as Māori (national average is 14%), high unemployment, low incomes and poor educational outcomes. Along with these heightened risk factors come levels of long-term conditions higher than the national averages. These factors indicate that Te Whiringa Ora is working in an area that faces significant challenges. These challenges would struggle to be addressed with a traditional general practice-based chronic care programme.

The profile of the patients enrolled in the programme (from February 2011 to December 2012) was 55% female; 49% Māori, 49% NZ European, 2% other; average age 69; and 62% referrals from the GP. The initial cohort targeted by the programme was 139 patients with COPD and having been admitted to hospital 2 or more times in the past 12 months and/or 6 primary care visits in the past 12 months (including Emergency Department visits). After the first few months, the target group was expanded to include all chronic diseases, with the focus on recruiting 20 new patients each month, to supporting a maximum of 550 patients per annum. This model assumed that the average time in the programme was 6 months (it was found to be 5 months).

The reason for the focus on high users of hospital inpatient services and ED was due to the resource implications of hospital and ED visits, and a belief that many of these visits could be avoided if the patient’s condition was better managed in the community. This is the thrust of much of the Better, Sooner, and More Convenient government policy. One that assumes more effective and integrated care in the community will reduce pressure on hospital resources [2].

The programme incorporates the principles of Whānau Ora meaning that the individual and their family/ whānau are involved as partners in care, for example when discussing care and treatment options or other goals-based objectives. These may relate to health outcomes (i.e. better self-management of their chronic illness), but could equally be related to addressing housing issues, employment issue, child care resources, family conflict, etc. In this way the programme takes an holistic view and provides for a focus on the full range of determinants of health [3], while still achieving the objectives outlined above.

Referral into the programme usually comes from the GPs (62% of all historical referrals) or the hospital, though over time an increasing number of referrals came from other community agencies and from self/family referrals. The referral includes the patient being given information about the programme, and a consenting process. If the client agrees to participate, then the referring agency makes contact with the Te Whiringa Ora programme office to make the referral. The programme office then makes a decision if the patient can be referred onto the programme, based on appropriateness (meeting the eligibility criteria).

While the criteria for accepting referrals has relaxed somewhat since the programme was introduced, the following core criteria are used to make referral decisions:

- the clients need to be enrolled with the local Primary Health Organisation and
- have been diagnosed as having two or more long-term conditions and/or
- have an exacerbation of a long-term condition and
- require intensive management of their illness and
- be identified as having high complex needs [i.e. 2 or more hospital admissions and/or 6 primary care visits in the past 12 months (including emergency department visits)].

### Approach to care

The core components of the service are outlined in the flowchart in Annex 1]. The core components of the service include:

- An assessment of need (annex the assessment tool).
- A series of home visits.
- Telephone monitoring where deemed necessary.
- Self-management support.
- Referrals to other social, community and health services.
- A shared care plan.
- Telehealth monitoring.

Patients who meet the entry criteria are identified by GPs, hospital staff, Whānau Ora providers, allied health staff and district nurses. They are then offered a referral to Te Whiringa Ora. If it is not the GP making the referral they are encouraged to inform the GP of the referral. The referrals are prioritised on the identified level of risk, and all referrals are acknowledged within two days of receipt.

Once the patient is referred, a case manager and the kaitautoko will visit the patient and their whānau. The case managers are registered nurses, while the kaitautoko are those in the community, with experience in community health or mental health. In addition, they all have well-developed community connections and extensive knowledge of Tikanga Māori. The role of the case manager is to bring clinical skills, while the kaitautoko provides cultural support and lifestyle coaching to assist the patient and their whānau to achieve their goals. The case manager and kaitautoko work as a team, with the average patient load of 60 patients per team.

It is important to note though that while the programme operates from the principles of Whānau Ora, the programme is not just for the Māori population. As mentioned earlier, roughly half those enrolled are non-Māori, and the outcomes for Māori versus non-Māori are not statistically different. Essentially, the principals of Whānau Ora are about patient self-determination of need, and the various health professionals (case manager and kaitautoko) are there to help them address their needs.

During the initial home visits the Flinders assessment tool is completed to identify the patient’s needs and care plan requirements. It may take up to five home visits for the whānau to agree on the outcomes they want to achieve and the care plan to be completed. Once completed, the GP is notified of the care plan and there is often phone or face-to-face discussion. The case manager and kaitautoko then work with the patient and their family/whānau, the general practice team and other service providers to help meet the targets identified in the shared care plan.

A component of the programme is the possibility for the patient to have a telehealth monitoring unit in their home. The measurements include heart rate, blood pressure (BP), spirometry, pulse oximetry, body temperature, body weight and blood glucose levels. However, the system data are accessible by clinical staff so they can pick up early signs of exacerbation. The system also encourages self-management through learning to monitor their symptoms and better manage their condition and seek help at the appropriate times

Rather than this being used as a long-term monitoring tool, it has been found that the telehealth units act as a powerful self-management training tool over a shorter period of time. This is achieved by improving a patient’s ability to identify deterioration in their condition and then acting appropriately to address it. This insight in the programme lead to the team to develop a pre-screening selection tool to identify suitable telehealth patients, based on their likelihood of improving their self-management ability over a shorter period of time (see Annex 2)

## Part 2: implementation and organisation

### Implementation

Initial planning for the programme began early 2010 with consultation amongst clinicians, allied health and iwi stakeholders. This phase took a little over 9 months.

Before rolling out the programme it was vital to get the engagement and support of the hospital-based staff and the GPs. This is a factor found to be important in many studies of integrated care programmes [4]. The other key factor of course was the recruitment and training of key programme staff - particularly the kaitautoko. Engagement building was developed through the consultation and development phase. However, as detailed below, once the programme was rolled out it was found that significant resources needed to be put into relationship development and ‘selling’ the programme.

The implementation process was, unsurprisingly, not without challenges. These were particularly marked in the Eastern Bay of Plenty with the high patient needs, large geographic spread and existing services under immense pressure. The challenges to implementation can be grouped into four areas: perceived duplication of existing services, patient engagement, practice engagement and planning challenges.

When initially implemented, Te Whiringa Ora was perceived to be very similar to another programme provided by another service in the community. This led to debate and confusion amongst programme staff and healthcare professionals as to the role of the two services. This was resolved over time, with the realisation that Te Whiringa Ora provided case management based on an assessment followed by navigation, whereas the other service provided for coordination and service navigation. The other difference was that Te Whiringa Ora was focused on complex patients with a higher level of risk than the other service.

Patient engagement was a further challenge, as is often the case in programmes that are provided outside of the general practice [5]. The main challenge was due to the degree of unplanned (and unresourced) patient follow up that was required to get patient engagement. For example, letters were sent out for patients to consent to be enrolled. If there was no subsequent follow up by the practice staff to the patient (via phone) then enrolments were lower - particularly amongst Māori.

While these two challenges were manageable, the challenge of practice engagement was far more resource intensive, and there is still evidence of practices not all being completely comfortable with the programme design. The first factor related to engagement in the implementation phase was the communication process to providers about the new programme. There was evidence of mass mail-outs being used to communicate about the new Te Whiringa Ora programme to patients. However, this lead to some confusion and anxiety amongst some providers and patients around losing existing services. This was exacerbated by local providers unsure of Te Whiringa Ora’s function, believing the service was taking their existing patients over. This concern and confusion was only alleviated with thorough one-to-one follow-up explanations.

A deeper issue effecting practice engagement related to the model being patient centred. While all practices prescribe to a patient centred perspective, the Te Whiringa Ora model is seen by some as potentially focusing on goals that they thought as secondary to improving health. For example, it is not uncommon in a Whānau Ora model for patients to want to address employment or educational goals before they focus on health-related issues (e.g. smoking or dieting). When these differing priorities emerge there is some evidence of providers wanting to take a more health centric position, and somewhat erode the patient-centred philosophy. In these cases, and throughout the programme more generally, the shift to a strong patient-centred focus was achieved though effective, ongoing, an individualised feedback to the practice about their patients. This was supplemented with regular communications between the Te Whiringa Ora staff, and the practice-based staff.

The third (and ongoing) factor related to the funding process. The funding for the programme was available due to ‘Care Plus’, Services to Improve Access, and Health Promotion (HP) funding being amalgamated and used more flexibly than the previous funding criteria allowed. This meant in some cases individual practices may have lost some Care Plus funding that went directly to their practices. Care Plus was a scheme in which practices were funded to provide free three monthly visits for patients with chronic disease enrolled in the programme. In responding to this concern the Te Whiringa Ora team reiterated that the patients were not losing access to support for chronic disease. Rather, it was simply being provided by another service. This would then free up the GPs/practice nurses to focus on providing for acute care - an area they were generally more comfortable in.

The fourth challenge relating to practice engagement in the implementation phase was developing trust between the GPs and the Te Whiringa Ora service team members. In the early stages of the implementation, the Te Whiringa Ora service had to build credibility with general practices. This was achieved by communicating the success through newsletters, and providing individual patient feedback to the GP. Te Whiringa Ora also used league tables to show patients referrals per practice. While crude, it did help increase practice engagement.

Relationship development was addressed by regular communication with face-to-face meetings with providers and practices. This was supplemented by a process to feedback regular patient summaries to GP practices, and the use of patient case studies. These were short stories of the progress the patient was making - from the patient perspective (see Annex 3). These were included in newsletters that were sent out to providers every 6 weeks.

To further support GP engagement the case managers and kaitautoko were allocated geographical areas and certain GP practices to ensure a degree of ownership of relationships was developed. These teams also shared a ‘relationship development plan’. It included key messages, quality of the relationship and any issues that arose from the contacts.

The final challenge for implementation was in the planning processes. Firstly, the service model was designed to initially focus on 139 COPD patients identified as meeting the entry criteria (based on the last 2 years of data). However, in a number of cases these patients had since died, moved on or were no longer appropriate. This meant that the initial targets could not be met, leading to an impact on the credibility of the programme in the eyes of the GPs and other health professionals. This was addressed by applying a dedicated resource to e-tag eligible patients within the current patient management system; by encouraging the general practices to identify eligible patients; and getting hospital-based staff to make the initial approach to patients on their ward, and getting the Te Whiringa Ora case manager to follow up.

The second unanticipated planning challenge was the amount of intensive training in the model, assessment tools and approaches, service manual, relationship building and confirmation of roles and responsibilities. The time required to do this meant the team was unable to service the required 30 patients per month - having a further impact on programme credibility. This establishment phase also identified significant input needed to confirm IT tool requirements and documentation processes. This highlighted the need to build in adequate time for training, and to stage the referral targets to ensure the programme expectations were met.

### Governance

The programme is owned, delivered and administered by Healthcare of New Zealand. They are a private company, and New Zealand’s largest provider of community-based health and disability support. However, the programme is delivered under contract through the Eastern Bay Primary Health Alliance. Under the concept of Alliance contracting, an alliance board was set up to govern the services funded by the Business Case (this is a separate entity to the Eastern Bay Primary Health Alliance Board of Directors). The key partners were Eastern Bay Primary Health Alliance, two providers (including Healthcare of New Zealand) and the local District Health Board. District Health Boards have responsibility for the planning and funding of all health services within their geographic boundaries. Each workstream within the Eastern Bay Primary Health Alliance’s business case (of which Te Whiringa Ora was one) had a service alliance leadership team established to govern the implementation of that initiative, and assist and provide guidance to ensure success. The Te Whiringa Ora SALT team was made up of five individuals (with a clinical and/or management background) from across the three agencies.

This group met via teleconference every two weeks for the first six months. Their function was to supervise the execution of the project deliverables to ensure targets are met. They are accountable for ‘go/no-go’ approval of major project milestones, maintaining project scope and terms of reference, approving risk and issue mitigation plans and approving changes to the programme. They were also responsible for conducting regular project reviews, broad organisational communication of the project value and impact, monitoring and reviewing project expenditure and managing contingency.

While these were the areas that the SALT was established for, the reality was that it was often a problem to get people to make the teleconference meetings. This is not a reflection of the support the individuals had for the programme. Rather, it is a reflection of the people having many clashing commitments and time pressures. Consequently, many of the more management focused tasks were handled by the Healthcare of New Zealand Te Whiringa Ora project board that was established. This was made up of Healthcare of New Zealand staff seconded to design and oversee the implementation of the project.

The monitoring role of the governance structure was aided by a reasonably robust performance measurement model. This is detailed in part 3 below. These measures were focused on fiscal sustainability, service capacity and client outcomes - and the details of these are addressed in part 3 of the case study. An evaluation framework was put in place at the beginning of the project to allow collection of data from the beginning with outcomes data being evaluated later in the project. Hence, different measures were developed over time.

### Organisation

The Te Whiringa Ora service is owned by Healthcare of New Zealand Limited and was funded by the Eastern Bay Primary Health Alliance. As explained above, Eastern Bay Primary Health Alliance resulted from the amalgamation of three smaller Primary Health Organisations. Healthcare of New Zealand designed the programme, employs and manages the staff, and provides support for monitoring, the shared care platform and the telehealth units.

Multiple organisations are involved in the planning and delivery of Te Whiringa Ora. Hence, the range of care practices from Eastern Bay Primary Health Alliance provides referrals, ongoing clinical links and are obviously key in the discharge process. The District Health Board also sits on this alliance and they represent two interest groups - the funding for all publicly funded health care in the region, and the interests of the acute hospital. It is through this representation that the acute hospital is engaged in the programme. They have significant interest in supporting a programme that reduces demand on the hospital services. As the district health board (DHB) directly (or indirectly) funds the local health care system, there is a financial incentive to engage, as well as a desire to ensure quality care for patients. There are, or course, a number of other agencies involved in providing care (through referral) to help patients and their whānau address the goals in their care plan. These agencies are not contracted directly from Te Whiringa Ora, but are usually other government funded agencies that are aligned to address Whānau Ora^5^ goals.

**Figure 2.**
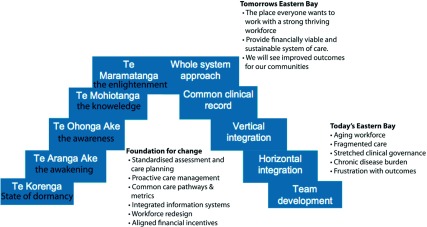
The values and drivers behind TWO in the Eastern Bay of Plenty

While the structural arrangements are important to understand, perhaps more important (and unique to New Zealand) is the strong cultural value base that makes the programme patient/whānau centred. As the service is founded on Whānau Ora principles, the planning and design are all about achieving whānau’s ultimate well-being - including cultural, spiritual, relational, environmental and economic dimensions. Alternatively, in Māori, people’s Mana Atua, Mana Whenua, Mana Tupuna, and Mana Tangata. The pathway to do this is illustrated in Figure 2.

The values on the left side relate to the journey Māori^6^ can take from an awakening (Te Aranga Ake) to Te Maramatanga (enlightenment). This can only be achieved through ensuring Māori participation, removing barriers to access, building Whānau, Hapu and Iwi capacity and increasing workforce development and capability. A key component here is iwi socio-economic development. This can be aided in the health system by moving from a siloed model to one that has a whole of system focus, with the patient and their family/whānau at the centre.

As the model highlights, integration across agencies is key. Te Whiringa Ora has developed a couple of ways to do this. Firstly, a shared care platform (within the Goldcare client management system) was developed (by Healthcare of New Zealand) to support cross-professional interaction. This was a new initiative in the region. While all health professionals in the community has access to the platform (but not hospital-based staff), there was a struggle to get effective engagement. One of the main reasons was that each professional had their own client management system to record notes, so the shared care platform was seen as an ‘extra’. Further, Goldcare was incompatible with the CMS system used by the GPs, and unable to communicate across platforms. Finally, the shared care platform is only available while the client is enrolled in Te Whiringa Ora (3–6 months). All these reasons compounded to the shared care platform not being utilised to the level intended.

The other important technological component is the telehealth monitoring unit (again provided by Healthcare of New Zealand). This allows the case manager (and the patient) to identify when clinical markers start to show signs of an eminent acute episode and intervene early. This also means that the case manager can communicate with the patients GP and relevant secondary care professionals to liaise around the patients care. Generally established clinical tolerance levels specific to that client helps them to identify what was normal for them and what as outside of the norms. From that basis the case managers can then link actions to be taken if measurements are outside of expected boundaries, i.e. medications. They unit also allows the case management and patient to link in the feeling/physiology associated with outside the norm recordings. This reduces the need for the monitor over time as the clients start recognising their signs and symptoms degrading.

The final key ‘organisational’ component relates to the staffing. The staff resource for the programme includes 2 FTE case managers, 2.5 FTE kaitautoko, 0.75 FTE administrator and 1 FTE service manager. The case managers are both registered nurses due to the needed clinical knowledge and oversight, whereas the kaitautoko have been selected because of their strong cultural competence - particularly in Tikanga Māori as well as community connections, social work experience and can do attitude.^7^ The patient would normally see their case manager and their kaitautoko, with the case manager providing assessments, clinical oversight and coordination, and the kaitautoko having more of a focus on engaging with clients, building trust through open communication of what was happening throughout the process, assisting and supporting with social, relational, emotional, financial and motivational needs.

The kaitautoko received a training package consisting of seven modules across 54 hours. It covered theoretical components that were explored through a workbook application of case studies, activities and assessment questions. Specific areas included an understanding of the service model, the roles and responsibilities of the staff, motivational interviewing, use of the shared care platform, understanding clinical data and an understanding of the local history of the people.

### Context

As mentioned above, funding for the business case and therefore Te Whiringa Ora was achieved by pooling several existing funding streams including ‘Care Plus’ funding that each practice had access for supporting patients with chronic illness(s). Without this, the initiative would not be feasible as the patients would not be able to pay for the service. It is important to remember that this community has the lowest level of socio-economic status (on a 10-point scale) and high levels of unemployment. Consequently, payment for services that relates to an individual’s health often comes at a lower priority that food and housing costs.

This means that the services accessed by the patients are almost free, with funding coordinated either at a regional-level through the District Health Board^8^ or at a national-level through the government. Funding for these services is from the tax revenue the government receives.

## Part 3: impact and sustainability

### Evidence of impact

The programme was designed by Healthcare of New Zealand with the view of rolling it out nationally if shown to be successful. Consequently, there were a large number of quantitative and qualitative performance measures established to help monitor the programme. These measures are outlined in Table 1, and collectively illustrate the goals and outcomes of the programme for the respective stakeholders.

These measures were explored through a formative evaluation conducted 6 months after the programme began, and then through a summative evaluation in the middle of 2012. Both of these evaluations documents have informed this case study. Since then, a value for money (cost-minimisation study) has also been conducted.

The initial cohort of 53 COPD patients was the focus of the summative evaluation. It is these patients that had been through the programme (average length of stay 4.5 months) and had 12 months of post-programme data to evaluate the impact against. The evaluation measured inpatient bed days, time between inpatient events and length of stay for this cohort against a control group.

#### Inpatient utilisation

For the cohort of 53 patients initially enrolled in the programme, the number of COPD bed days used in the 12 months prior to enrolment was some 40% more than 12 months after (see Table 2). For this same cohort of 53 their non-COPD bed days increased by 16%. Comparing the year pre-enrolment and post-enrolment the largest increases were seen in skin disorders, the nervous system and circulatory system.

#### Time between inpatient events

When comparing the time between COPD events for the year PRIOR to enrolment for both the first cohort of 53 and second cohort of 133 Te Whiringa Ora enrolees, their utilisation is very similar (see the chart below of the distribution of the time between events).

Once enrolled, the initial cohort of 53 Te Whiringa Ora patients has much longer between COPD events compared to the second cohorts utilisation in the year prior to enrolment (first graph) and their own pre-enrolment utilisation (second graph). The average cohort 1 patient would go 121 days between COPD events prior to enrolment but go 300 days between admissions post enrolment. That is, they gain an additional 179 admission free days or go less than half as frequently to hospital for COPD.


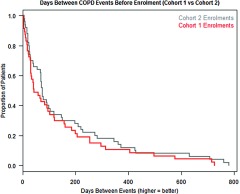


##### Length of stay – all events

When looking at all inpatient events after enrolment (and not just COPD events) there are no significant differences in length of stay for the first cohort (next two graphs).


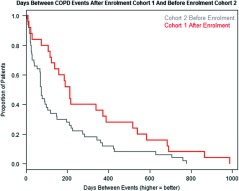



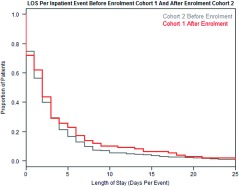



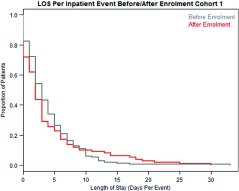


#### Impact on physicians

Qualitative and anecdotal data from the health professionals indicate that the programme is generally seen as positive, although with some early reservations (as noted earlier). GPs find the programme helpful as it provides a service for their clients that they cannot within the tightly control framework of the standard 10 min consultation process. They also realise that they often do not have the level of cultural context understanding that is needed to help address deep behavioural and social aspects that are impacting on the patients’ health. Finally, the programme helps the patients’ access services and resources that the GP often struggles to find (or be aware of). These factors contribute to the GPs involved in the programme seeing it as an effective option for their patients.

#### Value for money

In a more recent evaluation of the Te Whiringa Ora scheme, an economic analysis by Synergia Ltd. (funded by Healthcare of New Zealand) estimated that the project’s ‘net’ savings over five years for a community of 50,000 people was NZ$6.8 million with a potential to ‘break-even’ within 12 months (see Table 3). The savings calculation was derived from an understanding of the reduced costs (specifically in terms of hospital admissions) to clients in the Te Whiringa Ora programme compared against the predicted increase in costs within other similar localities without Te Whiringa Ora-type services. Synergia’s analysis also provided evidence for improved quality of care and improvement in health and equity suggesting that Te Whiringa Ora has gone some way to meeting the Triple Aim goals for quality improvement [6].

### Sustainability and spread

There are three main areas that affect the sustainability of the programme - funding, engagement and staffing.

As mentioned previously, the funding was achieved via reallocating several funding streams including Care Plus which each practice may have received to provide four free visits for patients with chronic disease (this fund was only used to 35% of its potential in the Eastern Bay). This initially raised some concerns within the practices that did utilise this funding, but most saw the benefit to the patient so were supportive. However, one group of practices in the region followed a strong corporate model with a focus on profit maximisation. This led to this group of practices trying to move to another Primary Health Organistion - one that allowed them to have access to their proportion of the Care Plus money. This provided a major risk to the business case, and Te Whiringa Ora as the loss of this group of practices (roughly 30% of the total), and therefore 30% of the funding, would struggle to maintain and grow the programme without access to another funding stream. However, the district health board thwarted this move by not supporting a fourth PHO coming into their area.

The challenges in the implementation phase related to practice engagement which will need to be a continued focus if the programme is sustainable. Without referrals the programme cannot exist. This highlights the important of ongoing relationship development and feedback to the practices - particularly when there is turnover in the staff of the practice.

Like all organisational initiatives, they are only sustainable if there are effective - and this comes down to the quality and skills of the staff that the programme can attract and retain. Te Whiringa Ora has been successful so far in retaining their staff. However, labour market movements and changes in personal circumstances can quite easily lead to losing a staff member. Given there is just over 2 FTE in the case managers and the kaitautoko respectively, losing one member would have quite an impact on the effectiveness and sustainability.

These issues of sustainability of course are not unique to this part of New Zealand. In fact, they are common challenges to sustainability across the country and quite likely internationally. Given this, there is no particular reason for this programme not to be able to spread to other parts of the country. In fact, the intention of Healthcare of New Zealand is to take it to other parts of the country, if the evidence from the next planned summative evaluation illustrates the effectiveness of the programme. There are likely only two real barriers to implementing the programme in other parts of New Zealand. The first is the need for a champion in the place the programme is to be transferred to, and the other is a recognised need for the programme (i.e. there is not something already running for this target population). The challenge of perceived duplication is a likely problem - as it was in the Eastern Bay of Plenty. Many district health boards have initiatives in place for supporting people with long-term conditions. However, most of them are more traditional GP centric chronic care programmes. The trick in getting this initiative spread is selling the idea that a community-based initiative that has a strong network and good cultural support is more likely to be effective than one runs out of a GP clinic. This is not a small challenge given the funding levers that New Zealand (and other international countries) has created to make the GP the central point for supporting people with long-term conditions.

That said, the programme does highlight some important lessons for those overseas considering similar programmes. Firstly, not to underestimate the time that needs to be put in at the beginning (and throughout) to relationship development and engagement with the general practice staff. They need to trust the service is providing something that is valuable to the patients, and that there are effective outcomes. The second point highlighted in this programme is the importance of investing in staff who have strong cultural competence - and partnering them with those who have the clinical credibility. Just a focus on clinical skills (which is the dominant model) is unlikely to be relatable to patients whom have quite different worldviews and priorities.

### Ongoing adaptations to support sustainability

The formal reviews of the programme, and a more general quality improvement process, have identified a number areas that have led to programme adaptations. These are all intended to help increase the acceptance of the programme amongst physicians, and improve its impact on patient outcomes. The first area relates to resourcing. As a result of the initial phase of the programme there has been an increase in programme resourcing to support the time needed for staff to familiarise themselves with the shared care plan, and build relationships with the general practice team. The other resourcing adaptation was an increased focus on training needs for Te Whiringa Ora staff - particular for the kaitautoko.

The other adaptation made has been to the enrolments process. Firstly, rather than enrolling based on specific diseases, the focus has moved to enrolling being based on patients that will gain value and requires case management to help improve self-management. Secondly, the process to begin enrolments has been streamlined. This has included ensuring up to date hospital admission data are collected; a dedicated resource is e-tagging eligible patients within the Patient Management System; GPs being encouraged to discuss the service with patients who are eligible; and high-flyers (those attending ED 4 or more times in last 12 months) are identified within the general practice.

## Part 4: barriers and facilitators to effective implementation

### Systemic and contextual factors

There are three key systemic and contextual factors that have contributed to the impact of Te Whiringa Ora. The first is a couple of the policies of the national^9^ lead government. The first is the ‘Better, Sooner, More Convenient’ policy that provided more flexibility in the funding streams for the development on integrated care initiatives across the country. The second is the Whānau Ora policy which has encouraged (and funded) agencies to work with Whānau to help identify and work towards achieving their goals. These policies act as both a facilitator and a barrier. They are both aimed at supporting more holistic and integrated care, but the Better, Sooner, and More Convenient policy has much more defined expected outcomes. This has led to funders putting pressure on the Te Whiringa Ora services to illustrate how it is impacting on measures such as hospital utilisation. This creates a barrier to Māori self-determination as their focus may not be on health goals immediately, but rather something in other areas, e.g. housing or employment.

The second key contextual factor has been that Te Whiringa Ora has had a supportive district health board. The Bay of Plenty district health board, of which Eastern Bay of Plenty covers one half, has been facing immense pressure on its hospital service in the region. Consequently, anything in the community that has the potential to reduce hospital demand they are interested in and supportive of.

A third key factor is that the history of the relationship between the alliance partners has been good, especially between the three primary health care organisations prior to aligning. This has meant the decision-making processes about Te Whiringa Ora have not been as problematic as they otherwise may have been since a degree of trusts and understanding was already present. However, there remain some tensions in the partnership - for example, between partners who are providers versus those who are funders - and that staff changes over time have meant that historical relationships and understanding of the original purpose of Te Whiringa Ora have sometimes become lost in more current decision-making.

### Organisational factors

A key strength of the programme is the Whānau Ora values that drive decision-making. These values mean that local communities are more likely to engage in the service, and they are more patient centred. However, these values can create challenges in delivering services effectively, particularly if ‘effectively’ is defined from the perspective of different stakeholders. From a funder position, an effective service is one who is efficient, improves clinical outcomes and reduces demand on the secondary service. From a patient and family/whānau perspective it may take somewhat longer to achieve these aims. This means the time taken with patients and their family can be quite long and the desired impact on secondary care resource utilisation may not be seen immediately.

Aside from culture, the integrated aspect of the initiative is likely an important aid. This integration is seen in the alliance structure used, the programme design that encourages multidisciplinary interaction and the information technologies (shared care platform and telehealth unit) that support agencies to be connected up to one another through the patient. This integration is aided at an individual level through the regular individualised feedback to general practices about patient progress. In this way, while the Te Whiringa Ora team provides much of the patient care, the GP still feels engaged. This factor is vital for when the patient is discharged from the programme (after 3–6 months) back into the care of their GP. The regular communication also helps alleviate concerns about duplication of services, and a perception that the programme means that the GP loses care for the patient.

The third organisational factor is the well-developed performance measurement system. This aids effective services delivery because the case managers and kaitautoko can track the programme at a population level, and make adjustments accordingly. It also facilitates GP and hospital staff engagement as the system provides evidence about the impact of the programme at a patient level and a system level. It also provided the ‘funders and governors’ with evidence this service was working.

### Operational/service delivery factors

The founding principles of Whānau Ora also help explain why care coordination at the patient level is effective. Firstly, the use of the kaitautoko in the team means the patient and their whānau has someone they can relate to and develop trust with. This is vital to a programme within this community with 50% of the population being Māori.

The service model was also very strong in engaging patients and their whānau in shared decision-making. The use of a goals-based approach to care enabled far greater involvement and pro-active involvement from people in the management of their care that has been a key aspect to Te Whiringa Ora’s success. For example, whānau are always invited to be a part of the plan, but the level of involvement is always left to the discretion of the client. When whānau outcomes are advanced, the whānau as a whole becomes stronger. This enables them to better able to support the member of their family who has the chronic illness(s) - a key factor in people being able to self-manage [7].

The other important operational design factor is the strong focus on multidisciplinary decision-making within the patients’ home. This means that the multiple issues that are related to the effective management of the patients’ chronic illness(s) can be addressed collectively. This is likely more effective for the patient than having to repeat their story multiple times, and each provider focusing just on what their domain of expertise is (for example: clinical, cultural, social or pharmaceutical).

## Conclusions

Te Whiringa Ora is a programme that is trying to do something different for a very high needs population, in a context that has reducing secondary care resources. While the programme clearly has significant potential, the challenges in designing, developing and delivering the programme identify important learning for other national and international contexts. In designing the programme the Te Whiringa Ora team was careful to consult widely with primary care agencies, hospital services and iwi providers. Despite this extensive process, there was still considerable energy needed to be placed into relationship development and communication in the early stages of implementation. The extent of time this took was unanticipated and, consequently, not adequately resourced. This is likely a common implementation challenge in introducing integrated care programmes in other contexts, and planners should take note and resource this important part adequately. In developing the programme there was the obvious need to find a way to fund it. The pooling of several funding streams to allow some new models to be lead to some concern amongst the GPs as they perceived they were losing revenue or previously funded services. The challenge for the Te Whiringa Ora team was to illustrate as early as possible the impact the programme was having on patient outcomes. This was done though a good performance measurement system, and highlight patient stories in regular newsletters. The lesson here is that when funding is moved from one existing area to another it is vital that attention is given to measuring the impact of the new programme on things that matter to those that may have ‘lost’ the funding. Where Te Whiringa Ora was smart was using data early on (patient stories) while they developed the evidence base of clinical outcomes and hospital resource utilisation.

Despite these design and development challenges, Te Whiringa Ora has managed to introduce a service that has a couple of features that make it somewhat unique to New Zealand. Firstly, the development of the kaitautoko role is central in the programmes ability to engage patients and the family/whānau. This is absent in many other integrated programmes nationally and internationally. While they may have a navigator or care coordinator, they generally do not have someone in the team that can provide lifestyle coaching from a strong position of cultural competence. The second somewhat unique feature is the use of the shared care platform. This is important because many integrated care programmes do not have such a platform. The lesson from the Te Whiringa Ora service is that a shared care platform will take some time to get people to use freely - particularly if they continue to have access to any previous system they use to maintain patient notes.

Overall, the greatest strength of Te Whiringa Ora as a programme of care has been its commitment to the principles of Whānau Ora and self-management. These principles are about holistic, culturally sensitive and care that start from the desires of the patient and their family/whānau. Much of the research to date strongly supports using such a perspective in care management as a way to stimulate more activate patients and family members in self-care [8]. However, all too often, the care received in many other integrated care programmes is defined by the health system - Te Whiringa Ora is on a journey to change that.

## Figures and Tables

**Table 1.**
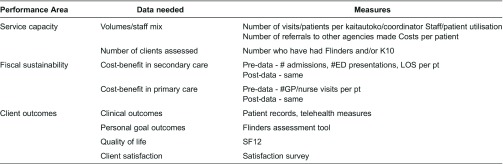
Performance measures in the TWO programme

**Table 2.**
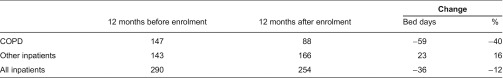
Total COPD bed days utilised the 12-month period before and after enrolment for the initial cohort of 53 patients with 12-month follow-up

**Table 3.**
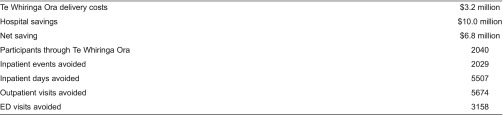
Projected five-year pilot costs and impacts of TWO

Source: King et al. [6], page 4.

## Notes

^1^​ Can be translated to mean ‘care connections’.

^2^​ Iwi means peoples, or sometimes understood as tribes. Across New Zealand there are approximately 200 iwi, a number of which are sub tribes of a larger tribe. There are 9 recognised Iwi in Eastern Bay of Plenty. Iwi are important groupings in New Zealand, and have responsibility for delivering a range of health, social, and educational services.

^3^​ Whānau is a Māori word that means extended family.

^4^​ Whānau Ora relates to the well-being of whānau and is determined by each whānau in relation to their circumstances. Whānau Ora is also the name of a number of New Zealand Government policies and strategies that are designed to inform service development and funding - particular in the health and social services sector. The implications of these strategies is that the outcomes that would be funded and support are, in part, those individual whānau collectively agree are what is important for them.

^5^​ Whānau Ora (discussed previously) is a New Zealand Government policy and set of strategies designed to inform service development and funding - particular in the health and social services sector. The implications of these strategies is that the outcomes that would be funded and support are, in part, those individual whānau collectively agree are what is important for them.

^6^​ While these are particular concepts form Māori, they are also actively used in the framework for working with non-Māori, i.e. a process from determining goals through to goal achievement and celebration.

^7^​ Refers to well-developed knowledge of general behaviour and guidelines for daily life and interaction in Māori culture

^8^​ A district health board is an entity that has overall responsibility for the funding and provision of all state funded health care in its region. There are 20 district health boards across New Zealand.

^9^​ The National Party is considered a centred right government and they entered into a coalition agreement with the Māori Party when they came into power in 2008.

## Annex 3. Patient Case Study sent in regular quarterly newsletter to general practices

This was a letter sent to TWO from a loving husband.

‘As I got home from hospital mid April 2012, after an operation, my dear wife Herma got sick with Alzheimer's disease. This was the start of a so trying period of our lives after living together for forty years. I myself in need of care became the lone carer for dear Herma for 24 hours a day for four months. Herma was going down fast, mentally and physically. And so was I. I was crying out for help, but to no avail! Herma was having one fall after another, till she broke her arm. Out of nowhere came help! Natasha and Jan in a selfless way were here to help us and for the first time I didn't feel alone anymore. There were no empty promises but actions from the word GO! This gave me hope and a good feeling because Herma was in good hands. Up to this time both of these ladies are helping us with practical advice and looking after Herma and me.

So, thank you, so much and a Merry Christmas to TWO from Christian and Herma’.

Herma was having frequent falls, many of these were at night, and as a result Christian would wake up to assist her up to six times per night. Herma's physical health was excellent; however, she suffers from the devastating effects of Alzheimer's disease. Unfortunately, Herma's cognitive abilities were deteriorating rapidly. This was a great concern to Christian as he was worried how he would be able to keep her safe, particularly as his own physical health was comprised by recent surgery and old injuries to his back. TWO received a referral from Radius practice nurse Oriwia Rehu-Murchie for Mrs Gross. An appointment was made to see Herma and Christian. After an explanation of the services TWO could offer, both Herma and Christian tentatively accepted TWO services. Radius Medical had identified the Gross's need for home support and an assessment for Support Net was completed by Oriwia. Sadly the application was denied as Mr and Mrs Gross did not have a community services card. Within two days two community services cards were issued to Mr and Mrs Gross through helpful guidance and support letters from the TWO team. This enabled the application for support to be processed by Support Net. Mr and Mrs Gross choose HCNZ as their service provider. As a result of hard work by HCNZ Service coordinator Sarah Wihapi, Natasha Manuel from TWO, Support Net and Oriwia Rehu-Murchie from Radius Medical Centre, home care support services commenced within a few days of referral. Herma was able to access temporary respite care as part of the package also Herma decided to take respite time in Golden Pond, as she had previously worked there making it familiar. Unfortunately, one week prior to Herma taking her respite care she suffered a fall and needed to be reviewed by Whakatane Hospital Emergency department which resulted in a fractured and dislocated wrist. Mr Gross found himself stranded at the hospital after riding alongside his wife in the ambulance and asked for help form TWO's Natasha Manuel. She arrived to assist the Gross's with a ride home and gave Christian help to settle Herma for the night. Christian struggled to cope that night and called for some support the following morning. It soon became obvious to Mr Gross and to all involved with their care that temporary respite for Mrs Gross was no longer an option, and Herma would be safer in long-term care. This was a hard decision especially for Christian, but in the end both Herma and Christian decided long-term care was the best option for them both. Christian now attends rehabilitation twice a week and no longer requires a crutch. The doting husband never fails to visit Herma daily in the rest home where she enjoys socialising and the organised activities.
